# Over-the-scope clip closure for afferent limb perforation during balloon enteroscopy-assisted endoscopic retrograde cholangiopancreatography in Roux-en-Y anatomy

**DOI:** 10.1055/a-2739-2343

**Published:** 2025-11-26

**Authors:** Kazuya Koizumi, Jun Kubota, Chihiro Sumida, Soichiro Nakaya, Makomo Makazu, Karen Kimura, Sakue Masuda

**Affiliations:** 1 13619 Gastroenterology Medicine Center, Shonan Kamakura General Hospital, Kamakura, Japan

Gastrointestinal perforation during endoscopic retrograde cholangiopancreatography (ERCP) is life-threatening 1 2 . The over-the-scope clip (OTS clip, Ovesco Endoscopy GmbH, Tübingen, Germany) has improved patient outcomes 3 . However, perforations during balloon enteroscopy-assisted ERCP (BE-ERCP) are challenging to close using the OTS clip because of anatomical constraints 4 . We report a case of afferent limb perforation during single (S) BE-ERCP in a patient with Roux-en-Y that was closed using the OTS clip delivered using a colonoscope (PCF-H290TI; Olympus Medical Systems, Tokyo, Japan; Video 1 ).

Successful closure of an afferent limb perforation with an over-the-scope clip using a colonoscope following balloon enteroscopy-assisted ERCP in a patient with Roux-en-Y anatomy.Video 1

A 75-year-old man with pancreaticoduodenectomy and Roux-en-Y for an intraductal papillary mucinous neoplasm underwent endoscopic ultrasound-guided liver abscess drainage. He later developed an intrahepatic bile duct stricture, confirmed using SBE-ERCP, unrelated to his prior disease or drainage. Bile cytology revealed mild atypia, and fluoroscopy-guided biopsy was negative for malignancy. SBE-ERCP was performed using a cholangioscope. Although not initially observed, a substantial amount of free air appeared later in the procedure ( Fig. 1 **a** and **b** ). A perforation of the afferent limb was also observed. Initial closure with hemostatic clips failed, worsening the leakage and perforation. The OTS clip was considered, and it required the removal of all placed clips, which further enlarged the perforation ( Fig. 2 **a** and Fig. 3 **a** ). Because the OTS clip actuation thread was too short for use with an enteroscope, a colonoscope was used. As the colonoscope with a distal attachment to the OTS clip could not pass because of jejunojejunostomy stenosis, balloon dilation was performed, and the scope was successfully advanced to the perforation site where the OTS clip was deployed, resulting in complete closure ( Fig. 2 **b** ). No contrast leakage was observed after OTS clip placement ( Fig. 3 **b** ). A biliary stent was placed through the intrahepatic bile duct stenosis, and a nasojejunal tube was placed in the afferent limb. The patient’s condition improved after conservative treatment and did not require further intervention. Adenocarcinoma was confirmed via cholangioscopy-guided biopsy.

**Fig. 1:**
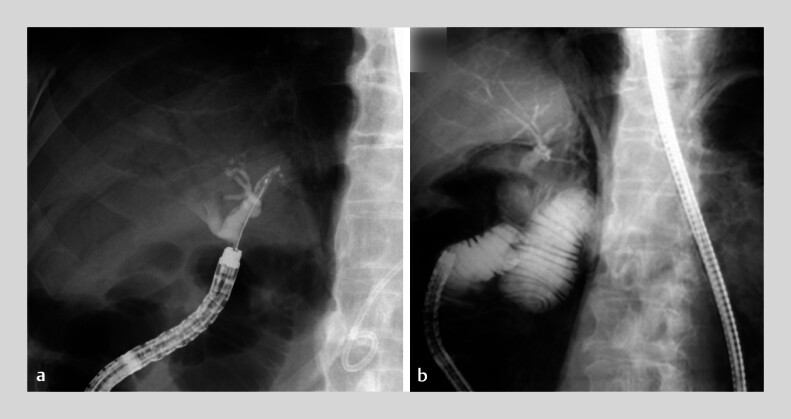
**a** No obvious free air was identified at the beginning of cholangiography during single-balloon enteroscopy-assisted endoscopic retrograde cholangiopancreatography in a patient with Roux-en-Y surgical anatomy. **b** Free air, initially absent, became evident during cholangioscopy.

**Fig. 2:**
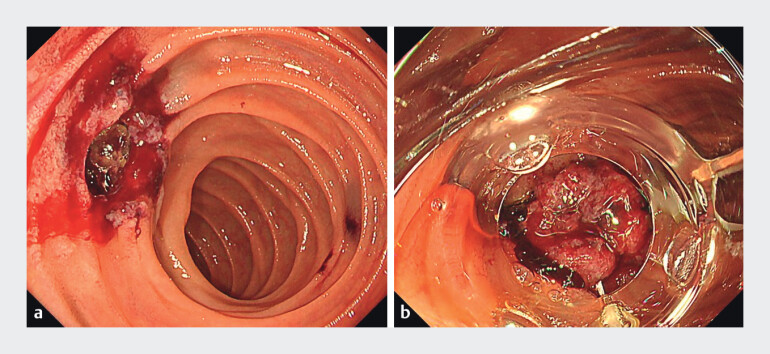
**a** Enlarged perforation in the afferent limb after the removal of the previously placed clip. **b** Successful closure of the afferent limb perforation with an over-the-scope clip using a colonoscope in a patient with Roux-en-Y surgical anatomy.

**Fig. 3:**
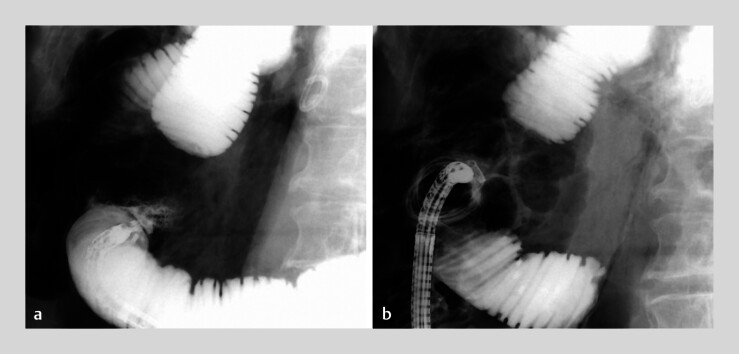
**a** Leakage of the contrast medium was observed from the perforation site in the afferent limb. **b** No contrast leakage was observed following over-the-scope clip placement.

Endoscopy_UCTN_Code_CPL_1AK_2AI

## Notes

Endoscopy E-Videos https://eref.thieme.de/e-videos
*E-Videos* is an open access online section of the journal *Endoscopy* , reporting on interesting cases and new techniques in gastroenterological endoscopy. All papers include a high-quality video and are published with a Creative Commons CC-BY license. Endoscopy E-Videos qualify for HINARI discounts and waivers and eligibility is automatically checked during the submission process. We grant 100% waivers to articles whose corresponding authors are based in Group A countries and 50% waivers to those who are based in Group B countries as classified by Research4Life (see: https://www.research4life.org/access/eligibility/ ). This section has its own submission website at https://mc.manuscriptcentral.com/e-videos .
